# The indicators for early blood transfusion in patients with placental abruption with intrauterine fetal death: a retrospective review

**DOI:** 10.1186/s12884-022-05187-9

**Published:** 2022-11-17

**Authors:** Yasuko Sano, Michi Kasai, Satoru Shinoda, Etsuko Miyagi, Shigeru Aoki

**Affiliations:** 1 Perinatal Center for Maternity and Neonates, Yokohama City University Medical Center, 4-57 Urafune-cho, Minami-ku, Yokohama, Kanagawa 232-0024 Japan; 2 Department of Biostatistics, Yokohama City University School of Medicine, Yokohama, Kanagawa 236-0004 Japan; 3 Department of Obstetrics and Gynecology, Yokohama City University School of Medicine, Yokohama, Kanagawa 236-0004 Japan

**Keywords:** Blood transfusion, Fetal death, Fibrinogen, Placental abruption, Pregnancy

## Abstract

**Background:**

Placental abruption (PA) with intrauterine fetal death (IUFD) is associated with a high risk of postpartum hemorrhage (PPH) resulting from severe disseminated intravascular coagulation (DIC). Therefore, blood products that are sufficient for coagulation factor replacement must be prepared, and delivery should occur at referral medical institutions that are equipped with sufficient blood products and emergency transfusion protocols. We retrospectively reviewed the records of patients with PA and IUFD (PA-IUFD) to identify possible factors that may indicate the need for early blood transfusion and investigated whether the Japanese scoring system for PPH can be applied in such cases.

**Methods:**

We used a database of 16,058 pregnant patients who delivered at Yokohama City University Medical Center between January 2000 and February 2016. Thirty-three patients were diagnosed with PA-IUFD before delivery and categorized into two groups–blood transfusion and non-transfusion–to compare the maternal characteristics and pregnancy outcomes.

**Results:**

In patients with PA-IUFD, the transfusion group exhibited significantly more blood loss; lower fibrinogen levels and platelet counts; higher levels of fibrin degradation products (FDP), D-dimer, and prothrombin time; and a tendency for tachycardia on admission, compared to the non-transfusion group. Many patients in the transfusion group had normal fibrinogen levels on admission but later displayed markedly decreased fibrinogen levels. The Japan Society of Obstetrics and Gynecology (JSOG) DIC score was significantly higher in the transfusion than in the non-transfusion group.

**Conclusions:**

In PA-IUFD, the fibrinogen level, platelet count, D-dimer, FDP, heart rate, and JSOG DIC score on admission may indicate the need for blood transfusion. However, even with normal fibrinogen levels on admission, continuous monitoring is indispensable for identifying progressive fibrinogen reductions in patients with PA-IUFD.

## Background

Placental abruption is a serious obstetric complication with high maternal and neonatal morbidity and mortality, occurring in 0.2–1% of pregnancies, a perinatal mortality rate of 3–12%, and an increased risk of maternal blood transfusion as well as death [1–6]. When a placental abruption is complicated by intrauterine fetal death (IUFD), it is defined as a severe abruption. Such a condition is associated with a high risk of postpartum hemorrhage (PPH) which can progress to severe disseminated intravascular coagulation (DIC) [3]. Therefore, it is necessary to manage such deliveries at referral medical institutions that are equipped with sufficient blood products and emergency transfusion protocols [7, 8]. PPH, a major cause of maternal mortality, has been reported to cause rapid consumptive or dilutional coagulopathy, especially in cases of placental abruption and amniotic fluid embolism [9–11].

Previous studies have identified blood fibrinogen level, Japan Society of Obstetrics and Gynecology (JSOG) DIC score [12], and shock index (SI) as indicators for blood transfusion in PPH [13]. A decrease in fibrinogen level has also been established as a pointer to the severity of PPH, placental abruption, and the need for blood transfusion [14, 15] However, factors that determine when placental abruption (PA) with IUFD (PA-IUFD) requires blood transfusion have not been identified. Therefore, we retrospectively reviewed factors indicating a requirement for early blood transfusion;i.e. initiation of transfusion before apparent/overt DIC and examined whether the same index as PPH can be useful in patients with PA-IUFD.

## Methods

### Study design

We retrospectively reviewed a database of 16,058 deliveries between January 2000 and February 2016 at the Yokohama City University Medical Center, a tertiary medical center. Patients diagnosed with PA-IUFD before delivery were extracted from the electronic medical records and included in this study.

Overall, 33 patients (0.21%) were diagnosed with PA-IUFD and were categorized into two groups: those who required (transfusion group; *n* = 22) and those who did not require (non-transfusion group; *n* = 11) blood transfusion. We compared maternal characteristics and pregnancy outcomes between the groups. Requirement for blood transfusion was decided by the attending physician.

We examined the maternal age, body mass index, gestational age at delivery, cesarean delivery rate, trial of vaginal delivery rate, time interval from hospital visit to delivery, primipara rate, birth weight, hypertensive disorders of pregnancy (HDP), small for gestational age (SGA), premature rupture of the membrane (PROM), hysterectomy, area of placental detachment, blood loss at delivery, blood loss until 24 hours after delivery, and the volume of each blood transfusion from onset to discharge (RBC, FFP, and platelet concentrates [PC]) as maternal characteristics and pregnancy outcomes. SGA was defined as a neonatal birth weight below the 10th percentile of the reference curves for birth weight for the gestational week in question. Blood loss at delivery was defined as blood loss within 2 h after delivery for both vaginal and cesarean deliveries. The fibrinogen level, shock index (SI), and JSOG DIC score on admission were compared between the two groups. Additionally, we compared and evaluated the maternal vital signs and laboratory data on admission—platelet count, prothrombin time (PT), activated partial thromboplastin time (APTT), fibrin degradation products (FDP), D-dimer, and hemoglobin (Hb)— between the two groups. We also examined the odds ratios (OR) for blood transfusion for each of the following factors: heart rate > 100 beats per minute (bpm), fibrinogen level of < 150 mg/dL or < 200 mg/dL, platelet counts < 10 × 10^4^ μL or < 20 × 10^4^ μL, and JSOG DIC score ≥ 7.

The correlation between FFP transfusion and each of the factors was also examined. For fibrinogen levels, sequential changes (on admission, minimum, and on the day after delivery) were also investigated case-wise.

### Statistical analysis

Results were expressed as median (range) and frequency (percentage) values. The JMP® version 15.0 was used for statistical analysis. Fisher’s exact test was performed for categorical variables and the Wilcoxon rank-sum test was used for continuous variables. Statistical tests bearing a *p*-value of < 0.05 were considered as significant. A 95% confidence interval (CI) was calculated for each OR.

## Results

In patients with PA-IUFD, the transfusion group exhibited significantly more blood loss; lower fibrinogen levels and platelet counts; higher FDP, D-dimer and PT; and a tendency for tachycardia on admission, compared to the non-transfusion group.

Regarding maternal characteristics and pregnancy outcomes, only blood loss was significantly higher in the transfusion than the non-transfusion group (Table 1). But, intriguingly, blood loss at and up to 24 h after delivery, and transfusion volume (RBC, FFP, PC), did not differ for vaginal delivery or cesarean section (median 1337 mL vs. 1650 mL; *p* = 0.52, 1395 mL vs. 1762 mL; *p* = 0.52, 6 U vs. 4 U; *p* = 0.87, 4 U vs. 4 U; *p* = 0.85, 0 U vs. 0 U; *p* = 0.64, respectively, data not shown).

**Table 1:** Maternal characteristics and pregnancy outcomes

	Transfusion group (*n* = 22)	Non-transfusion group (*n* = 11)	*p*-value
Maternal age (years)	33.5(22–40)	34(24–41)	0.76
Body mass index (kg/m^2^)	20.6(17.8–34.8) (*n* = 16)	19.2(17.7–24.7) (*n* = 9)	0.19
Gestational age at delivery (weeks)	33(22–40)	34(24–41)	0.89
Cesarean delivery (%)	16(73%)	7(64%)	0.70
Trial of vaginal delivery (%)	8(36%)	4(36%)	1.0
Time from hospital visit to delivery (minutes)	107.5(36–355)	88(31–305)	0.8
Primipara (%)	9(41%)	8(73%)	0.14
Birthweight(g)	1813(384–3428)	1588(1034–2748)	0.41
HDP (%)	7(32%)	3(27%)	0.71
SGA (%)	5(23%)	3(27%)	1.0
PROM	0	0	
Hysterectomy	1(5%)	0(0%)	1.0
Area of placental abruption(%)	100(40–100) (*n* = 21)	62.5(5–100) (*n* = 10)	0.016
Blood loss			
estimated blood loss on delivery (mL)	2084.5(212–4008)	1000(357–1472)	0.0003
estimated blood loss during 24 hours from delivery (mL)	2273(212–4014)	1050(357–1536)	0.0002
Transfusion			
RBC (U)	8(0–20)	0	< 0.00001
FFP (U)	8(0–34)	0	< 0.0001
PC(U)	0(0–40)	0	0.027

Results were expressed as medians (range) and frequency (percentage)

*HDP* Hypertensive disorders of pregnancy, *PROM* Premature rupture of membranes, *SGA* Neonatal birth weight below the 10th percentile of the reference curves of birth weight for the gestational week. *RBC* Red blood cells, *FFP* Freshly frozen plasma, *PC* Platelet concentrate

No significant differences were found for other maternal characteristics, including cesarean section rates and time from hospital admission to delivery. No patients died. There were no complications associated with transfusion, such as thrombosis and pulmonary edema.

For maternal laboratory data, vital signs, and JSOG DIC scores on admission, lower fibrinogen levels and platelet counts, higher D-dimer, FDP, PT levels and Japan Society of Obstetrics and Gynecology (JSOG) DIC score were found in the transfusion group (137 mg/dL vs. 292 mg/dL; *p* = 0.01, 10.8 × 10^4^/μL vs. 19.0 × 10^4^/μL; *p* = 0.009, 105.1 μg/dL vs. 5.9 μg/dL; *p* = 0.023, 338 μg/dL vs. 13.4 μg/dL; *p* = 0.02, 13.7 s vs. 11.9 s; *p* = 0.012, 8.5 vs. 7; *p* = 0.035, respectively, Table 2.

**Table 2:** Maternal data, vital signs, and JSOG DIC on admission

	Transfusion group(*n* = 22)	Non-transfusion group (*n* = 11)	*p*-value
Fibrinogen level (mg/dL)	136.5(30–391)	292(131–411)	0.01
Fibrinogen < 150 mg/dL	12(55%)	2(18%)	0.07
Fibrinogen< 200 mg/dL	14(64%)	3(27%)	0.07
Platelet count(×10^4^/μL)	10.8(1.5–23.8)	19(8–30.6)	0.009
Platelet count< 10 × 10^4^/μL	9(41%)	2(18%)	0.26
Platelet count < 20 × 10^4^/μL	21(95%)	7(63%)	0.03
PT (s)	13.7(10.1–44)	11.9(9.1–13.8) (*n* = 10)	0.012
APTT(s)	31.8(21–150)	29.5(22.3–40.9) ﻿(*n* =10)	0.34
FDP (μg/mL)	338(45.3–674) (*n* = 12)	13.4(6–124) (*n* = 3)	0.02
D-dimer (μg/mL)	105.1(7.6–306.4) (*n* = 10)	5.9(3–10) (*n* = 3)	0.023
Hb (mg/dL)	10.3(6.1–13.8) (*n* = 19)	11.3(8.8–12.8) (*n* = 10)	0.12
JSOG DIC score	8.5(6–17)	7(5–9)	0.035
HR (bpm)	85.5(63–137)	77(62–115)	0.08
HR > 100 bpm	8(36%)	1(9%)	0.21
sBP < 90 mmHg	2(9%)	1(9%)	1
SI	0.68(0.44–1.94)	0.55(0.45–0.91)	0.41

*PT* Prothrombin time, *APTT* Activated partial thromboplastin time, *FDP* Fibrin/fibrinogen degradation products, *Hb* Hemoglobin, *JSOG DIC* Japan Society of Obstetrics and Gynecology disseminated intravascular coagulation, *HR* Heart rate, *bpm* Beats per minute, *sBP* Systolic blood pressure, *SI* Shock index

The frequency of fibrinogen levels of < 150 mg/dL or < 200 mg/dL did not differ significantly (*p* = 0.07 and *p* = 0.07, respectively). The frequency of platelet counts < 20 × 10^4^/μL was significantly different (*p* = 0.03) compared to the non-transfusion group.

The maternal heart rate on admission was slightly faster in the transfusion group (85.5 bpm vs. 77 bpm; *p* = 0.08), but the proportion of maternal tachycardia (HR > 100 bpm) was not significantly higher (36% vs. 9%; *p* = 0.21) compared to the non-transfusion group. There was no significant difference in the SI (*p* = 0.41).

ORs (95% CI) for factors requiring transfusion were 4.7 (0.96–22.8) for fibrinogen levels < 200 mg/dL, 5.4 (0.94–31.0) for fibrinogen levels < 150 mg/dL, and 12 (1.14–126) for platelet counts < 20 × 10^4^/μL (Table 3).

**Table 3:** Odds ratios of factors requiring transfusion

	Odds ratio	95% CI
Fib< 150 mg/dL	5.4	0.94–31.0
Fib< 200 mg/dL	4.7	0.96–22.8
Platelet< 10 × 10^4^/μL	3.1	0.54–18.0
Platelet< 20 × 10^4^/μL	12	1.14–126
JSOG DIC score ≧7	2.2	0.49–10.6
HR > 100 bpm	5.7	0.61–53.2

*JSOG DIC* Japan Society of Obstetrics and Gynecology disseminated intravascular coagulation, *HR* Heart rate, *Fib* Fibrinogen

**Fig. 1:**
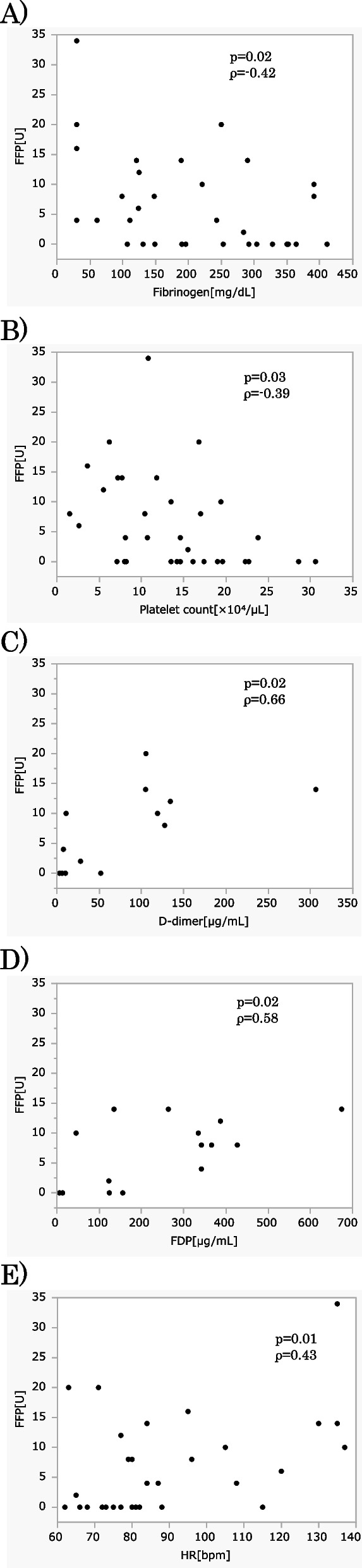
Correlation between FFP transfusion and each factor. **A** Correlation between FFP transfusion and fibrinogen level (mg/dL). **B** Correlation between FFP transfusion and platelet count (×10^4^/μL). **C** Correlation between FFP transfusion and D-dimer (μg/mL). **D** Correlation between FFP transfusion and FDP (μg/mL). **E** Correlation between FFP transfusion and heart rate. All laboratory and vital sign data are on admission

Figure 1 shows the correlation between the total unit of FFP transfusions and each factor. Fibrinogen levels and platelet counts were negatively correlated (ρ/*p* values were of − 0.42/0.02 and, − 0.39/0.03, respectively), while D-dimer, FDP, and heart rate were positively correlated with FFP transfusion (ρ/*p* values were of 0.66/0.02, 0.58/0.02, and 0.43/0.01, respectively).

**Fig. 2:**
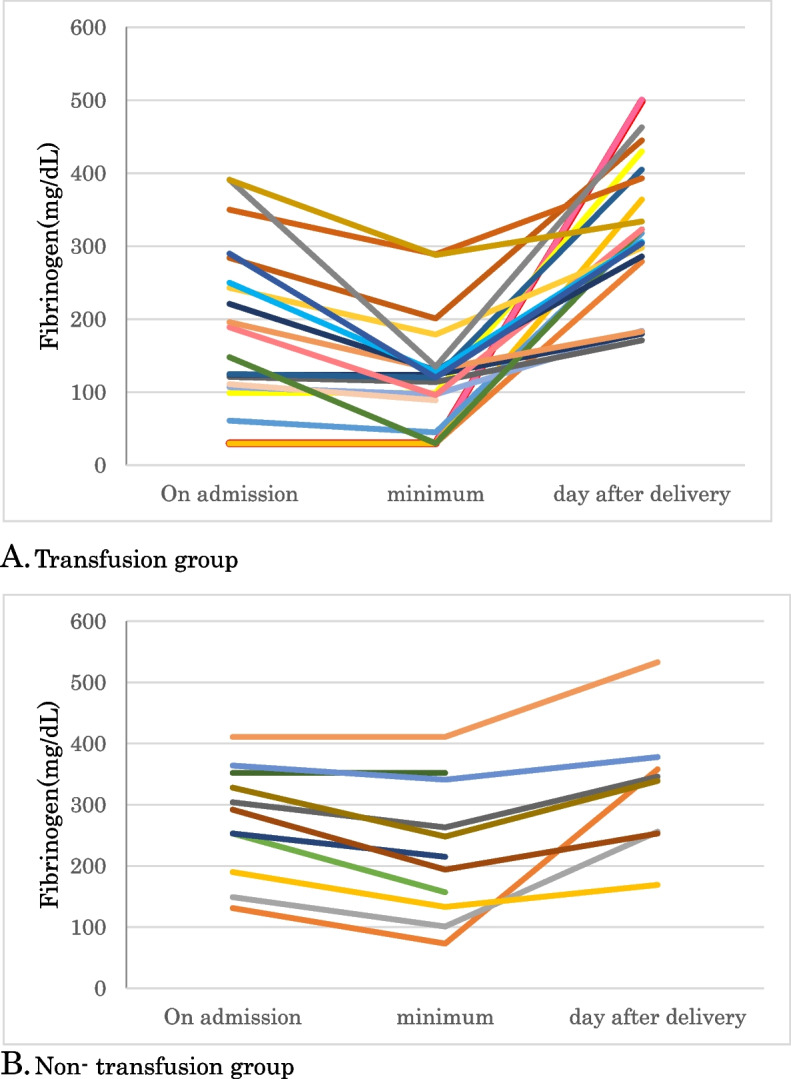
Changes in the fibrinogen levels observed between the two groups. **A** Transfusion group **B**) Non-transfusion group

Many patients in the transfusion group had normal fibrinogen levels on admission but later displayed markedly decreased fibrinogen levels (Fig. 2). All patients with fibrinogen levels < 100 mg/dL on admission required transfusion, and 14 of 16 patients with fibrinogen levels > 200 mg/dL on admission exhibited decreased fibrinogen levels after admission.

Even in patients with preserved fibrinogen levels (fibrinogen levels > 200 mg/dL), the platelet count on admission was lower, albeit not significantly, in the transfusion group than that in the non-transfusion group (14.5 × 10^4^/μL vs. 21.0 × 10^4^/μL; *p* = 0.06).

## Discussion

Our study bears three important implications. First, in patients with PA-IUFD, the transfusion group bore lower fibrinogen levels and platelet counts; higher FDP, D-dimer, and PT, a tendency for tachycardia on admission, and a higher total blood loss compared to the non-transfusion group. Second, even when fibrinogen levels were normal on admission, these levels markedly declined in many patients in the transfusion group later. Third, we concluded that the JSOG DIC score may be used in patients with PA-IUFD.

### On admission findings and blood loss

The transfusion group exhibited significantly lower fibrinogen and platelet counts, higher FDP, D-dimer, and PT on admission, and more blood loss compared to the non-transfusion group. It is not surprising that the transfusion group displayed more blood loss, but this was not associated with the mode or duration of delivery. Fibrinogen levels have been reported to be useful indicators of PPH severity and blood transfusion [13, 14]. In the present study, the ORs for blood transfusion were 5.4 and 4.7 for fibrinogen levels < 150 mg/dL and 200 mg/dL, respectively, suggesting that, in the cases with PA-IUFD, a fibrinogen level < 200 mg/dL could indicate a need for blood transfusion. In addition, a negative correlation was observed between fibrinogen levels and the total FFP transfusion volume, as reported by Matsunaga et al. for placental abruption cases [16]. Our data strongly support the importance of fibrinogen levels as an indicator for transfusion in PA-IUFD reported by Atallah et al. [17].

Interestingly, not only was the platelet count lower in the transfusion group, but also a count of 20 × 10^4^/μL or less had high ORs for transfusion, indicating that transfusion is more likely to be required in PA-IUFD, even for patients with normal to mildly low platelet counts, likely because platelet consumption is progressing due to consumptive coagulopathy associated with placental abruption [9].

Our data revealed FDP and D-dimer levels were correlated with FFP transfusion in PA-IUFD for the first time, although correlation between the severity of placental abruption and pre-delivery FDP and D-dimer values has been reported as a result of consumptive coagulopathy [7, 18]. Therefore, FDP and D-dimer levels may be useful indicators of the need for transfusion in PA-IUFD. In addition, the heart rate was positively correlated with FFP transfusion. The heart rate can be evaluated easily and quickly and, as tachycardia is associated with a decrease in the circulating blood volume, it may be a useful indicator for blood transfusion.

To the best of our knowledge, this is the first study to suggest a relationship between blood transfusion and heart rate for PPH, but our sample size was small; therefore, further studies are required. Intriguingly, the SI was not significantly different in this study. This may be because placental abruption is frequently complicated with HDP. The incidence of chronic hypertension (CH) and gestational hypertension in normal pregnancies in Japan have been reported to be 0.6–1.5% and 6–25%, respectively [19–22], but 1/4 of placental abruption is complicated by CH [23]. In the present study, HDP was observed in approximately 30% of the patients. This suggests that changes in heart rate may be more important than SI in PA-IUFD because a decrease in blood pressure in hypertensive women does not initially increase SI. PT was significantly prolonged in the transfusion group, but the difference was small and, in line with the study of Collins et al [24], possibly due to fibrinogen depletion. However, this small difference is expected to be insufficient for it to be used as an indicator.

### Changes in fibrinogen levels

Fibrinogen levels were markedly decreased after admission in the transfusion group. Although there were eight patients in the transfusion group with fibrinogen levels > 200 mg/dL on admission, fibrinogen levels subsequently decreased in all these patients. Furthermore, seven of the eight patients had decreased fibrinogen levels on admission despite receiving FFP transfusion, suggesting that their coagulopathy was progressive and severe. A low platelet count suggests a progressive decline in fibrinogen levels. In a comparison of patients with fibrinogen levels of > 200 mg/dL on admission between the transfusion and non-transfusion groups, the platelet count on admission was 14.5 × 10^4^/μL (7.1–23.8 × 10^4^/μL) in the transfusion group and 21.0 × 10^4^/μL (13.5–30.6 × 10^4^/μL) in the non-transfusion group, with a tendency to be lower, albeit not significantly, in the transfusion group (*p* = 0.06). We conclude that, even when the fibrinogen level is normal on admission, if the platelet count is low, there is a possibility of progressive and severe coagulopathy; therefore, serial monitoring of fibrinogen is essential in patients with PA-IUFD.

### JSOG DIC score

In Japan, the JSOG DIC score is used for the early diagnosis and treatment of PPH. It reflects clinical findings, vital signs, and laboratory data, and when it is ≥8 points, the patient is likely to progress to DIC [12].

We propose blood loss, heart rate, fibrinogen level, platelet count, D-dimer, and FDP as deciding factors for initiating transfusion in patients with PA-IUFD, all of which are included in the JSOG DIC score. The JSOG DIC score was significantly higher in the transfusion than the non-transfusion group, highlighting the usefulness of the JSOG DIC score in PA-IUFD.

### Limitations

This study has two major limitations. First, prior to 2016, our hospital did not have a standardized protocol for blood transfusions and DIC treatment. Treatment was decided by the attending physician, and therefore, the treatment strategies varied. A massive transfusion protocol (MTP) was published in the field of trauma in 2007 and has been used in obstetrics in recent years, but is not yet established. A fresh frozen plasma (FFP)/red blood cell (RBC) ratio > 1 is suggested [25–27] There were no strict protocols for blood transfusion yet, and transfusion was decided by the attending physician, consisting mainly of RBC transfusion or an RBC:FFP ratio of approximately 1:1 based on the amount of blood loss and other indicators. Under these circumstances, comparing the two groups was challenging.

Second, the number of cases was low. Although multivariate analysis is appropriate for detecting blood transfusion factors, we could only perform a univariate analysis. Moreover, because of the low number of cases, there were many factors for which the Fisher’s exact test showed no significant difference. To address these shortcomings, further studies will be required.

## Conclusion

In placental abruption with IUFD, fibrinogen levels, platelet count, heart rate, D-dimer, FDP, PT, and JSOG DIC score on admission may all be useful indicators for transfusion initiation. However, even when the fibrinogen level is within the normal range on admission, serial monitoring of fibrinogen is essential, as the latter can progressively decrease in patients with PA-IUFD.

## Data Availability

The datasets used or analysed during the current study are not publicly available as the low cases may allow for identification and be a breach of confidentiality but are available from the corresponding author on reasonable request.

## Abbreviations

IUFD: Intrauterine fetal death
PA: Placental abruption
PA-IUFD: Placental abruption (PA) with IUFD
PPH: Postpartum hemorrhage
DIC: Disseminated intravascular coagulation
JSOG: Japan Society of Obstetrics and Gynecology
SI: Shock index
HDP: Hypertensive disorders of pregnancy
SGA: Small for gestational age
PROM: Premature rupture of the membrane
RBC: Red blood cells
FFP: Fresh frozen plasma
PC: Platelet concentrates
PT: Prothrombin time
APTT: Activated partial thromboplastin time
FDP: Fibrin degradation products
Hb: Hemoglobin
OR: Odds ratios
CH: Chronic hypertension

## References

[1] Ananth CV, Keyes KM, Hamilton A, Gissler M, Wu C, Liu S (2015). An international contrast of rates of placental abruption: an age-period-cohort analysis. PLoS One.

[2] Oyelese Y, Ananth CV (2006). Placental abruption. Obstet Gynecol.

[3] Ananth CV, Lavery JA, Vintzileos AM, Skupski DW, Varner M (2016). Severe placental abruption: clinical definition and associations with maternal complications. Am J Obstet Gynecol.

[4] Ruiter L, Ravelli AC, de Graaf IM, Mol BW, Pajkrt E (2015). Incidence and recurrence rate of placental abruption: a longitudinal linked national cohort study in the Netherlands. Am J Obstet Gynecol.

[5] Ananth CV, Berkowitz GS, Savitz DA, Lapinski RH (1999). Placental abruption and adverse perinatal outcomes. JAMA.

[6] Thachil J, Toh CH (2009). Disseminated intravascular coagulation in obstetric disorders and its acute haematological management. Blood Rev.

[7] Erez O, Mastrolia SA, Thachil J (2015). Disseminated intravascular coagulation in pregnancy: insights in pathophysiology, diagnosis and management. Am J Obstet Gynecol.

[8] Honda M, Matsunaga S, Era S, Takai Y, Baba K, Seki H (2014). Intrapartum anti-disseminated intravascular coagulation therapy leading to successful vaginal delivery following intrauterine fetal death caused by placental abruption: a case report. J Med Case Rep.

[9] Rattray DD, O'Connell CM, Baskett TF (2012). Acute disseminated intravascular coagulation in obstetrics: a tertiary Centre population review (1980 to 2009). J Obstet Gynaecol Can.

[10] Santoso JT, Saunders BA, Grosshart K (2005). Massive blood loss and transfusion in obstetrics and gynecology. Obstet Gynecol Surv.

[11] Goffman D, Nathan L, Chazotte C (2016). Obstetric hemorrhage: a global review. Semin Perinatol.

[12] Kobayashi T (2014). Obstetrical disseminated intravascular coagulation score. J Obstet Gynaecol Res.

[13] Era S, Matsunaga S, Matsumura H, Murayama Y, Takai Y, Seki H (2015). Usefulness of shock indicators for determining the need for blood transfusion after massive obstetric hemorrhage. J Obstet Gynaecol Res.

[14] Charbit B, Mandelbrot L, Samain E, Baron G, Haddaoui B, Keita H (2007). The decrease of fibrinogen is an early predictor of the severity of postpartum hemorrhage. J Thromb Haemost.

[15] Wang L, Matsunaga S, Mikami Y, Takai Y, Terui K, Seki H (2016). Pre-delivery fibrinogen predicts adverse maternal or neonatal outcomes in patients with placental abruption. J Obstet Gynaecol Res.

[16] Matsunaga S, Seki H, Ono Y, Matsumura H, Murayama Y, Takai Y (2012). A retrospective analysis of transfusion management for obstetric hemorrhage in a Japanese obstetric center. ISRN Obstet Gynecol.

[17] Atallah A, Piccin G, Dubernard G, Abdul-Hay MJ, Cortet M, Huissoud C. Fibrinogen for the prediction of severe maternal complications in placental abruption with fetal death after 24 weeks of gestation. Int J Gynaecol Obstet. 2022. 10.1002/ijgo.14417 Online ahead of print. 10.1002/ijgo.14417 PMC10087485 35986606

[18] Su J, Yang Y, Cao Y, Yin Z (2021). The predictive value of pre-delivery laboratory test results for the severity of placental abruption and pregnancy outcome. Placenta.

[19] Yoder SR, Thornburg LL, Bisognano JD (2009). Hypertension in pregnancy and women of childbearing age. Am J Med.

[20] Hauth JC, Ewell MG, Levine RJ, Esterlitz JR, Sibai B, Curet LB (2000). Pregnancy outcomes in healthy nulliparas who developed hypertension. Calcium for preeclampsia prevention study group. Obstet Gynecol.

[21] Bateman BT, Bansil P, Hernandez-Diaz S, Mhyre JM, Callaghan WM, Kuklina EV (2012). Prevalence, trends, and outcomes of chronic hypertension: a nationwide sample of delivery admissions. Am J Obstet Gynecol.

[22] Ananth CV, Duzyj CM, Yadava S, Schwebel M, Tita ATN, Joseph KS (2019). Changes in the prevalence of chronic hypertension in pregnancy, United States, 1970 to 2010. Hypertension..

[23] Pritchard JA, Cunningham FG, Pritchard SA, Mason RA (1991). On reducing the frequency of severe abruptio placentae. Am J Obstet Gynecol.

[24] Collins PW, Lilley G, Bruynseels D, Laurent DB, Cannings-John R, Precious E (2014). Fibrin-based clot formation as an early and rapid biomarker for progression of postpartum hemorrhage: a prospective study. Blood.

[25] Miyata S, Itakura A, Ueda Y, Usui A, Okita Y, Ohnishi Y (2019). Transfusion guidelines for patients with massive bleeding. Japanese J Transfusion Cell Ther.

[26] Royal College of Obstetricians and Gynaecologists (2016). Prevention and management of postpartum haemorrhage (green-top guideline no. 52). BJOG.

[27] Borgman MA, Spinella PC, Perkins JG, Grathwohl KW, Repine T, Beekley AC (2007). The ratio of blood products transfused affects mortality in patients receiving massive transfusions at a combat support hospital. J Trauma.

